# Corneal power modelling with OCT data – Thin and thick lens paraxial models versus raytracing

**DOI:** 10.1111/aos.70052

**Published:** 2025-12-02

**Authors:** Achim Langenbucher, Nóra Szentmáry, Alan Cayless, Peter Hoffmann, Jascha Wendelstein

**Affiliations:** ^1^ Department of Experimental Ophthalmology Saarland University Homburg (Saar) Germany; ^2^ Department of Ophthalmology Semmelweis‐University Budapest Hungary; ^3^ School of Physical Sciences The Open University Milton Keynes UK; ^4^ Augen‐ und Laserklinik Castrop‐Rauxel Castrop‐Rauxel Germany; ^5^ IROC AG, Institut für Refraktive und Ophthalmo‐Chirurgie Zurich Switzerland; ^6^ Department of Ophthalmology Ludwig‐Maximilian‐University Munich Munich Germany

**Keywords:** corneal power, keratometry, multivariable linear model, paraxial thick cornea model, raytracing, Monte Carlo simulation

## Abstract

**Background:**

Evaluating keratometric power with Zeiss index (PKZ), paraxial thick cornea power (Gullstrand [PG]) and power referenced to the front (PFV) and back vertex plane (PBV) and raytracing power (PR), and modelling the deviation from PKZ with a multivariable linear prediction model.

**Methods:**

A dataset of 4604 Casia2 measurements from a cataractous population was Copula expanded to *N* = 30 000 maintaining the individual univariate distributions and interactions. PKZ was compared with paraxial thick lens corneal power PG, PFV, PBV, and PR using measured pupil size and variations of apertures from 1 to 6 mm.

**Results:**

On average, PKZ/PG/PFV/PBV was 43.05/42.74/42.83/43.59 D, and PR was 43.03 D with the measured pupil size. Varying pupil size from 1 (1) 6 mm increased mean PR with aperture size (42.84/42.91/43.02/43.17/43.37/43.62 D). The multivariate linear models predicting the deviation of PG‐PKZ, PFV‐PKZ, and PBV‐PKZ with corneal radii and central thickness performed well, with *R* ^2^ = 0.93 and a root mean squared prediction error of 0.01 D, whereas the equivalent model for PR‐PKZ with corneal radii and asphericities, corneal thickness, and aperture sizes performed less well, with *R* ^2^ = 0.79 and a root mean squared prediction error of 0.18 D.

**Conclusion:**

Corneal power derived using the paraxial thick cornea model differs from keratometric power and a linear model performs well in predicting the differences. However, raytracing power differs even more from keratometry with the linear model far less effective.

## 1 INTRODUCTION

In daily clinical routine, the ‘real’ corneal power measure is of minor interest, and ophthalmologists focus mainly on changes in corneal power over time or laterality (Langenbucher et al., 1998; Langenbucher, Szentmáry, et al., 2022; Schröder et al., 2018). However, the refractive power of the cornea is crucial for predicting the refractive outcome of the patient when calculating optical implants, for example, in the planning of cataract surgery or refractive lens procedures and in refractive corneal surgery (Preussner et al., 2003; Schröder et al., 2018).

Since keratometers do not measure corneal power directly, keratometer indices are routinely used to convert the measured curvature of the corneal front surface to corneal power. Various keratometer indices have been established in the past including the Zeiss index (nK = 1.332) or the Javal index (nK = 1.3375), and each keratometer index generates its own corneal power values for the same corneal curvature measurement (Langenbucher, Szentmáry, Weisensee, Cayless, et al., 2021; Langenbucher, Szentmáry, Weisensee, Wendelstein, et al., 2021; Preussner et al., 2003). Neither manual nor automated keratometry, nor Placido topography provides access to central corneal curvature (Douthwaite, 2003; Navarro et al., 2006, 2019). Most of the tomographers based on Scheimpflug or optical coherence tomography (OCT) technology also provide ‘simulated keratometry data’ derived from the mid‐periphery for consistency with manual or automated keratometers.

We know that the cornea is a convex–concave meniscus lens with two predominantly aspherical surfaces (Liou & Brennan, 1997; Navarro et al., 2006, 2019). However (automated), keratometry is restricted to measurement of corneal front surface radius (e.g. in the flat and steep meridians) (Preussner et al., 2003) and does not take account of asphericity. Placido topography allows for measuring the shape of the corneal front surface including asphericity, and tomography can determine curvature (in the flat and steep meridians, together with surface asphericities) for both the corneal front and back surfaces, additionally measuring corneal thickness (Navarro et al., 2006, 2019).

There are various definitions of corneal power, including keratometric power derived from the corneal front surface curvature using a keratometer index, paraxial total corneal power considering corneal front and back surface curvature and thickness referenced either to the secondary principal plane or to the front or back vertex plane, and raytracing total corneal power based on corneal front and back surface curvature, asphericity, and thickness referenced, for example, to the corneal front vertex plane derived from the ray focus or to the wavefront focus position (Atchison & Cooke, 2024; Douthwaite, 2003; Iskander et al., 2001; Langenbucher et al., 1998; Navarro et al., 2019; Schröder et al., 2018).

The purpose of this study was

- To derive the keratometric power (based on a thin lens paraxial corneal model), the paraxial corneal power based on a thick lens corneal model, and the raytracing corneal power based on a thick lens corneal model considering asphericity and an aperture stop from tomographic measures.
- To back‐calculate the keratometer index which best converts corneal front surface curvature to the various corneal power values.
- To implement multivariable linear models in terms of a Monte Carlo simulation to predict the keratometer index and the differences between the various corneal power values and the keratometric power.

in a dataset containing anterior segment OCT data extracted from the Casia2 with measurements from a cataractous population.

## 2 MATERIALS AND METHODS

### 2.1 Dataset for our data analysis

In this retrospective study, a data download containing 5224 measurements from the Augen‐ und Laserklinik Castrop‐Rauxel, Castrop‐Rauxel, Germany was assessed. The local ethics committee (Ärztekammer des Saarlandes) provided a waiver for this study (157/21). The data were filtered at the source to include only measurements made prior to cataract surgery. Duplicate measurements of eyes were discarded from the dataset. Where measurements of both eyes were available, one eye was selected randomly for consideration in our evaluation. After filtering, the raw export data (.CSV‐format) were transferred to us in an anonymised fashion, precluding back‐tracing of the patient. The anonymised data contained tomographic measurements acquired using the Casia2 (Tomey GmbH, Nürnberg, Germany, software version Ver.50.5A.03). The CSV data were imported into MATLAB (Matlab 2024a, MathWorks, Natick, USA) for further processing.

### 2.2 Preprocessing of the data

Custom software was written in Matlab. The data exported by the Casia2 software included corneal front surface curvature (Ra1 in mm in the flat meridian (oriented at an angle of Aa1 in degrees); Ra2 in mm in the steep meridian (orientation Aa2 in degrees)) and asphericity derived in the central 6 mm zone (Qa), corneal back surface curvature (Rp1 in mm in the flat meridian (orientation Ap1 in degrees); Rp2 in mm in the steep meridian (orientation Ap2 in degrees)), central corneal thickness (CCT in μm), and the entrance pupil size (Pup in mm). Each data entry also contained quality markers QS from the Casia2 classifying the corneal front surface, back surface, CCT, and Pup values as ‘OK’, ‘Suspicious’ or ‘Error’. Data with a quality marker other than ‘OK’ for any of the above‐mentioned parameters, incomplete data, data with an ectasia screening marker other than ‘0%’ and measurements with a pupil size of larger than 4.6 mm were discarded from the dataset. The mean corneal front and back surface radii (Ra and Rp respectively) were calculated from the harmonic means of the respective curvatures in the flat and steep meridians (Ra1 and Ra2 or Rp1 and Rp2) (Langenbucher, Szentmáry, et al., 2022; Langenbucher, Szentmáry, Weisensee, Wendelstein, et al., 2021). The dataset was condensed to the relevant parameters Ra, Qa, Rp, Qp, CCT, and Pup.

### 2.3 Copulas for increasing the size of the dataset

In the next step, we expanded the dataset size for the Monte Carlo simulation and modelling. For that purpose, we constructed Copulas from the original dataset which maintain both the marginal univariate distributions of Ra, Qa, Rp, Qp, CCT, and Pup as well as the dependencies between the variables in our multivariate correlated dataset (Chen et al., 2023; Choi & Seo, 2022; Langenbucher, Schrecker, et al., 2022). Univariate kernel distributions were derived for all variables in the original dataset and the original data transformed to the Copula scale (multivariate uniform distribution) using a kernel estimator of the cumulative distribution function (Langenbucher, Schrecker, et al., 2022). From the transformed dataset, we then derived the Kendall's τ correlations between the variables and generated a random six‐dimensional Copula with a multivariate uniform distribution, having *N* = 30 000 datapoints and the Kendall's *τ* values derived from the transformed original dataset (Chen et al., 2023; Langenbucher, Schrecker, et al., 2022). In the last step, we transformed the multivariate Copula back using the original scale of the data and using the kernel estimator for the inverse cumulative distribution function. This expanded dataset was used for both the Monte Carlo simulation and the modelling (Chen et al., 2023; Choi & Seo, 2022; Langenbucher, Schrecker, et al., 2022).

### 2.4 Data processing and raytracing

The keratometric power (PKZ/PKJ in dioptres (D)) was calculated using the Zeiss (nK = 1.332)/Javal (nK = 1.3375) keratometer index with PKZ/PKJ = (nK‐1)/Ra. The surface power for the corneal front surface (Pa) and back surface (Pp) was derived with Pa = (nC‐1)/Ra and Pp = (nA‐nC)/Rp using the refractive indices for cornea (nC = 1.376) and aqueous humour (nA = 1.336) from the Liou‐Brennan schematic model eye (Liou & Brennan, 1997).

The paraxial corneal power with respect to the principal plane on the corneal image side was extracted using the Gullstrand formula as: PG = Pa + Pp‐Pa·Pp·CCT/nC. The paraxial corneal power referenced to the corneal back vertex plane (PBV, back vertex power) was calculated using the classical vergence formula as PBV = Pa/(1‐Pa·CCT/nC) + Pp, and the paraxial corneal power referenced to the corneal front vertex plane was derived from PBV as PFV = PBV/(1 + PBV·CCT/nA) (Liou & Brennan, 1997; Navarro et al., 2006, 2019).

We then used 2D raytracing to extract the power of the thick lens cornea model taking into account the surface asphericities for the measured pupil size Pup and also for pupil sizes from 1 to 6 mm in steps of 1 mm (in total 7 pupil sizes) (Clement et al., 1987; Langenbucher et al., 2011; Langenbucher, Szentmáry, et al., 2022; Langenbucher, Szentmáry, Weisensee, Cayless, et al., 2021; Langenbucher, Szentmáry, Weisensee, Wendelstein, et al., 2021). We used an optical model incorporating an aperture stop at corneal front vertex plane (at *x* = 0), an aspheric corneal front surface (with Ra and Qa) with its apex located at (x, y = 0), and an aspheric corneal back surface (with Rp and Qp) with its apex located at (x, y = CCT, 0) (Clement et al., 1987; Langenbucher, Szentmáry, et al., 2022). A bundle of 1000 collimated rays was constructed (equidistant on a square root scale $\sqrt{y}$ to account for area correction in terms of equidistant rays in the 3D case) (Langenbucher et al., 2011; Langenbucher, Szentmáry, et al., 2022; Langenbucher, Szentmáry, Weisensee, Cayless, et al., 2021; Langenbucher, Szentmáry, Weisensee, Wendelstein, et al., 2021). After implementation of Snell's law in vectorial form, the ray bundle was traced through the aperture and the two corneal surfaces, and the ray focus (bundle focus defined as the plane (*x* = xRF) with the least root mean squared (RMS) ray scatter) and the wavefront focus (plane (*x* = xWF) with the least RMS optical path length differences) were calculated (Clement et al., 1987; Langenbucher, Szentmáry, Weisensee, Wendelstein, et al., 2021). From the location of the ray focus and the wavefront focus, the raytracing corneal power (PR for the ray focus and PW for the wavefront focus) was derived as the refractive index nA divided by the distance between origin and xRF or xWF (PR = nA/xRF and PW = nA/xWF). In addition, we recorded the overall RMS wavefront error (rmsWFE) and the spherical aberration coefficient (Z40) by evaluating the optical path length differences of the rays at the wavefront focus xWF (Atchison et al., 2016; Atchison & Cooke, 2024; Douthwaite, 2003; Langenbucher et al., 2011; Langenbucher, Szentmáry, et al., 2022; Sicam et al., 2006).

For all corneal power values (PG, PFV, PBV, PR, PW; except for the keratometric power PKZ and PKJ where we used nK as preset), we back‐calculated the corresponding keratometer index (nKG = Ra·PG + 1, nKFV = Ra·PFV + 1, nKBV = Ra·PBV + 1, nKR = Ra·PR + 1, nKW = Ra·PW + 1).

### 2.5 Monte Carlo modelling and statistics

Linear multivariate correction models were defined for the differences between the paraxial power values PG, PFV, PBV, and the keratometric power PKZ. These included all parameters used for the paraxial calculations as predictors (Ra, Rp, and CCT). Similar models were also defined for the differences between the raytracing power values PR, PW, and the keratometric power PKZ. These included all parameters used for the raytracing calculations as predictors (Ra, Qa, Rp, Qp, CCT, and Pup/preset aperture sizes) (Chen et al., 2023; Choi & Seo, 2022; Langenbucher, Szentmáry, Weisensee, Wendelstein, et al., 2021). These models were optimised by minimising the RMS model fit error. In addition, we defined multivariate prediction models for the keratometer indices nKG, nKFV, and nKBV using Ra, Rp, and CCT as predictors and for nKR, nKW with Ra, Qa, Rp, Qp, CCT, and Pup/preset aperture sizes as predictors.

Descriptive data for the original dataset and the Copula expanded dataset (Chen et al., 2023; Choi & Seo, 2022; Langenbucher, Schrecker, et al., 2022) were summarised in terms of arithmetic mean, standard deviation (SD), median, and the lower and upper boundaries of the 95% confidence interval (2.5% and 97.5% quantiles). Boxplots were used to show the distributions for the corneal power values and the back‐calculated keratometer indices with their median (solid line in the box), interquartile range (lower and upper box boundaries), and 95% confidence intervals (lower and upper whiskers). Scatterplots were used to show the multivariate linear model predictions and the model fit errors as a function of the respective observations.

## 3 RESULTS

From the *N* = 5224 measurements from the Casia2 tomographer transferred to us, a total of *N* = 4604 were used after eliminating measurements with incomplete data or with a quality check other than ‘OK’. Table 1 shows the descriptive data for Ra, Qa, Rp, Qp, CCT, and Pup for the original dataset in the upper part and for the Copula expanded dataset containing *N* = 30 000 datapoints in the lower part.

**TABLE 1:** Explorative data of corneal front surface radius (Ra) and asphericity (Qa), back surface radius (Rp) and asphericity (Qp), central corneal thickness (CCT), and size of the entrance pupil (Pup).

		Ra in mm	Qa	Rp in mm	Qp	CCT in μm	Pup in mm
Dataset with *N* = 4604	Mean	7.7223	−0.2218	6.5376	−0.3219	547.53	3.0650
	SD	0.2706	0.1836	0.2608	0.2006	33.34	0.4785
	Median	7.7099	−0.2230	6.5370	−0.3356	547.50	3.1200
	2.5% quantile	7.2040	−0.6367	6.0371	−0.6968	483.38	2.1465
	97.5% quantile	8.2823	0.1672	7.0490	0.0605	611.60	3.8614
Copula expanded dataset with *N* = 30 000	Mean	7.7218	−0.2230	6.5373	−0.3207	547.37	3.0673
	SD	0.2731	0.1858	0.2641	0.2051	34.08	0.4893
	Median	7.7153	−0.2253	0.5359	−0.3348	547.41	3.1164
	2.5% quantile	7.1966	−0.6460	6.0269	−0.7029	480.30	2.1315
	97.5% quantile	8.2786	0.1654	7.0531	0.0730	612.91	3.8955

*Note*: Mean/SD/median/2.5% quantile/97.5% quantile refer to the arithmetic mean/standard deviation/median/lower/upper boundary of the 95% confidence interval. The upper part of the table relates to the original dataset, and the lower part to the Copula expanded dataset.

**FIGURE 1:**
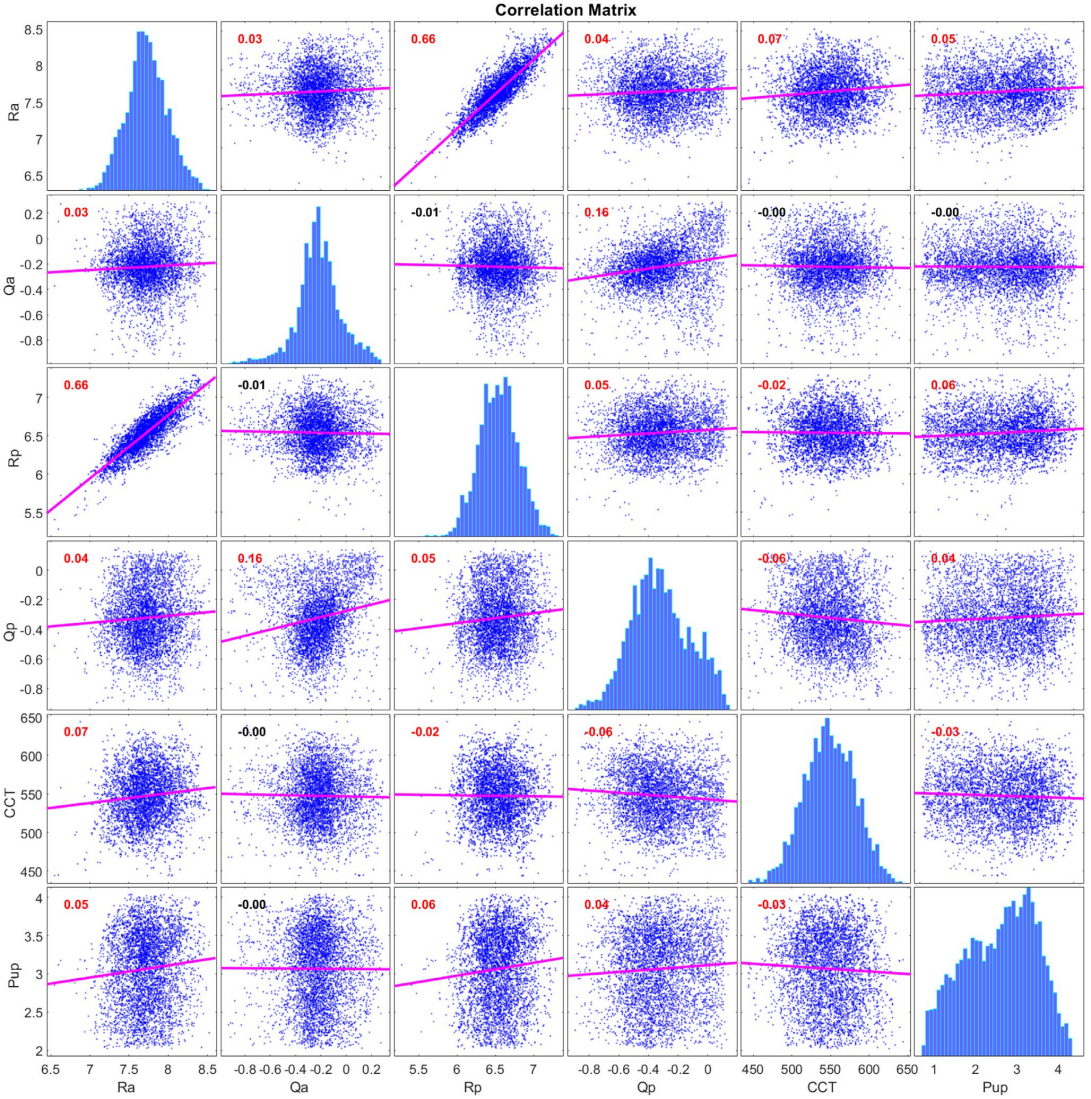
Correlation matrix plot showing the distributions (histograms on the diagonal) and the interactions (off‐diagonal graphs) between the corneal parameters based on the *N* = 4604 measurements. Ra and Rp refer to the corneal front and back surface radii in mm, Qa and Qp to the front and back surface asphericities, CCT to the corneal thickness in μm, and Pup to the measured size of the entrance pupil. The numbers shown in the top left corners of each graph refer to the Kendall's τ correlation coefficient, with statistically significant values highlighted in red. The magenta line on each graph refers to the corresponding regression.

Figure 1 displays the distributions (histograms on the diagonal) and the correlations between the variables (off‐diagonal scattergraphs) for the *N* = 4604 datapoints. The Kendall's τ correlation coefficients used to quantify the interactions between the variables for the Copula expansion of the dataset (Langenbucher, Szentmáry, et al., 2022) are shown in the respective scattergraphs (statistically significant correlations are marked in red).

Tables 2a and 2b lists the descriptive data for the corneal power values and the back‐calculated keratometer index for keratometry and paraxial calculations (Table 2a) and for raytracing calculations (Table 2b) for Pup and for variations of the aperture size from 1 to 6 mm in steps of 1 mm. The corneal spherical aberration in terms of the Z40 coefficient was on average 0.0149 ± 0.0138 μm for Pup and 0.0001 ± 0.0001 μm/0.0023 ± 0.0014 μm/0.0120 ± 0.0074 μm/0.0387 ± 0.0238 μm/0.0966 ± 0.0599 μm/0.2062 ± 0.1291 μm for aperture sizes 1/2/3/4/5/6 mm.

**TABLE 2A:** Corneal power values derived from the Copula samples: PKZ and PKJ refer to the keratometric power calculated with the Zeiss and Javal keratometer indices respectively, PG and nKG to the corneal power and back‐calculated keratometer index derived from the Gullstrand thick lens formula, PFV and nKFV to the corneal power and back‐calculated keratometer index for a thin replacement lens located at the front vertex plane, and PBV and nKBV to the corneal power and back‐calculated keratometer index for a thin replacement lens located at the back vertex plane of the cornea.

*N* = 30 000 copula samples	Keratometric		Thick cornea Gullstrand		Thick cornea front vertex		Thick cornea back vertex	
	PKZ in D	PKJ in D	PG in D	nKG	PFV in D	nKFV	PBV in D	nKBV
Mean	43.0492	43.7624	42.7447	1.3280	42.8253	1.3286	43.5908	1.3345
SD	1.5262	1.5515	1.5261	0.0009	1.5309	0.0009	1.5808	0.0010
Median	43.0316	43.7444	42.7320	1.3280	42.8122	1.3286	43.5759	1.3345
2.5% quantile	40.1033	40.7676	39.7949	1.3261	39.8639	1.3268	40.5302	1.3325
97.5% quantile	46.1327	46.8969	45.8369	1.3298	45.9228	1.3304	46.7690	1.3365

*Note*: Mean/SD/median/2.5% quantile/97.5% quantile refer to the arithmetic mean/standard deviation/median/lower/upper boundary of the 95% confidence interval.

**TABLE 2B:** Corneal power values derived from raytracing using the Copula samples as a function of pupil size (either the measured pupil size Pup or apertures varied from 1 to 6 mm in diameter at the corneal front apex plane): PKR and PKW refer to the corneal power extracted from the ray focus and wavefront focus respectively, and nKR and nKW to the corresponding back‐calculated keratometer index from the ray focus and wavefront focus.

*N* = 30 000 copula samples	Aperture size in mm➔	Pup (measured)	1	2	3	4	5	6
Raytracing corneal power PR in D	Mean	41.0284	42.8462	42.9092	42.0155	43.1668	43.3659	43.6169
	SD	1.5479	1.5326	1.5385	1.5596	1.5727	1.6101	1.6703
	Median	43.0138	42.8340	42.8997	43.0065	43.1533	43.3405	43.5876
	2.5% quantile	40.0440	39.8823	39.9394	40.0305	40.1453	40.2870	40.4483
	97.5% quantile	46.1534	45.9424	46.0169	46.1393	46.3489	46.6429	47.0353
Back‐calculated keratometer index nKR	Mean	1.3302	1.3288	1.3293	1.3301	1.3312	1.3328	1.3347
	SD	0.0014	0.0009	0.0010	0.0012	0.0017	0.0026	0.0037
	Median	1.3301	1.3288	1.3293	1.3301	1.3312	1.3327	1.3346
	2.5% quantile	1.3275	1.3269	1.3273	1.3276	1.3276	1.3272	1.3266
	97.5% quantile	1.3331	1.3305	1.3311	1.3324	1.3348	1.3381	1.3426
Raytracing corneal power PW in D	Mean	42.9773	42.8410	42.8882	42.9677	43.0806	43.2287	43.4145
	SD	1.5423	1.5322	1.5364	1.5448	1.5595	1.5834	1.6209
	Median	42.9613	42.8283	42.8778	42.9575	43.0684	43.2120	43.3872
	2.5% quantile	39.9984	39.8782	39.9228	39.9928	40.0797	40.1904	40.3163
	97.5% quantile	46.9808	45.9267	45.9888	46.0803	46.2258	46.4318	46.7133
Back‐calculated keratometer index nKW	Mean	1.3298	1.3287	1.3291	1.3297	1.3306	1.3317	1.3331
	SD	0.0012	0.0009	0.0009	0.0011	0.0014	0.0020	0.0028
	Median	1.3298	1.3288	1.3291	1.3297	1.3306	1.3317	1.3331
	2.5% quantile	1.3275	1.3269	1.3272	1.3275	1.3276	1.3275	1.3270
	97.5% quantile	1.3322	1.3305	1.3309	1.3318	1.3334	1.3358	1.3390

*Note*: Mean/SD/median/2.5% quantile/97.5% quantile refer to the arithmetic mean/standard deviation/median/lower/upper boundary of the 95% confidence interval.

**FIGURE 2:**
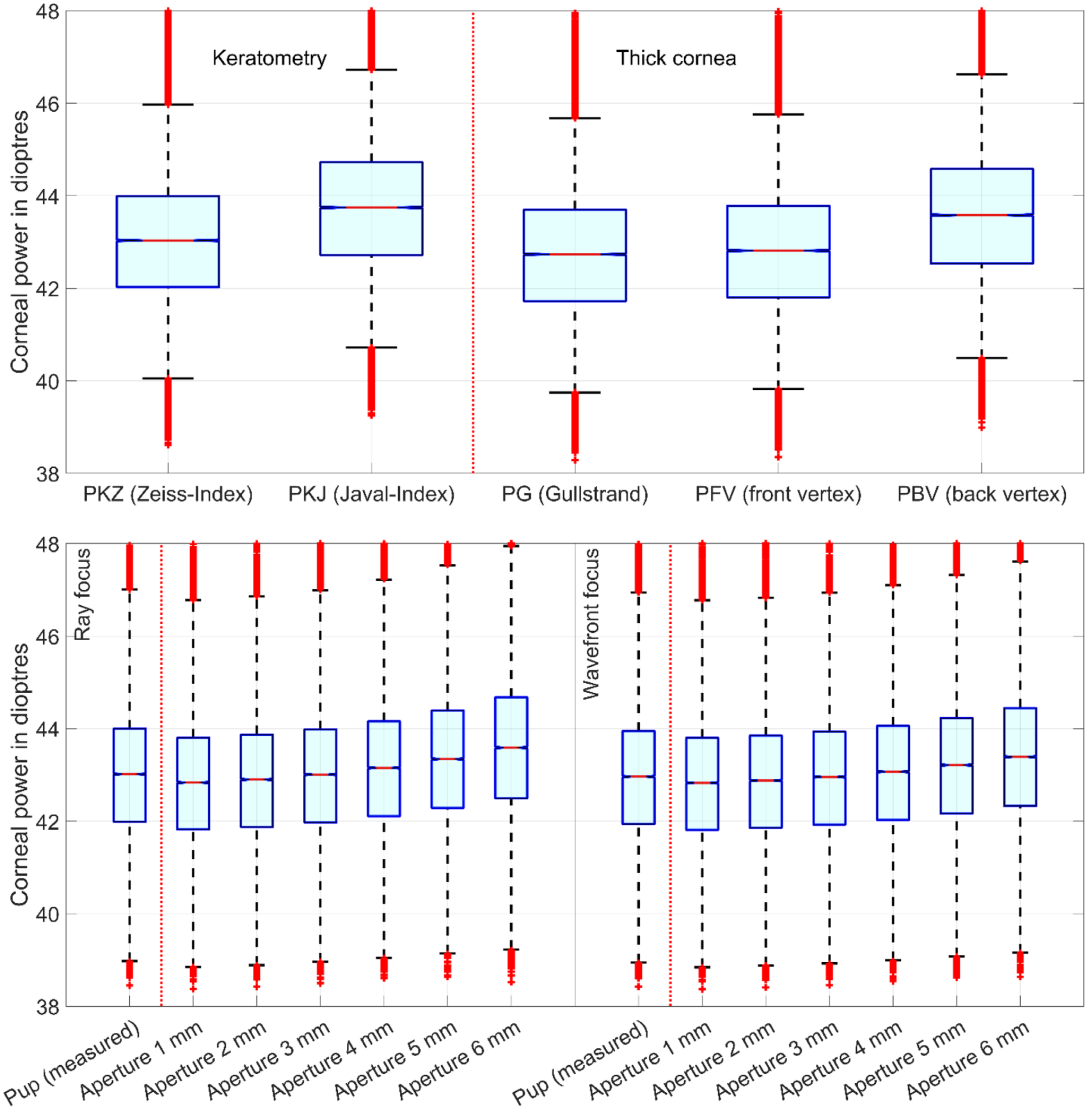
Corneal power as calculated from the Copula expanded dataset (*N* = 30 000). Upper graph: Keratometry (PKZ with Zeiss and PKJ with Javal keratometer index), paraxial calculations based on a thick lens cornea model (PG: Gullstrand power referenced to the image‐sided principal plane, PFV: Power referenced to the corneal front apex plane, PBV: Power referenced to the corneal back vertex plane). Lower graph: Raytracing calculations (PR: Ray focus, PW: Wavefront focus) derived with the measured pupil size (Pup) and with apertures from 1 to 6 mm in steps of 1 mm. This graph indicates that the corneal power increases with the aperture size as a result of the typically positive spherical aberration of the cornea. The raytracing corneal power generally shows a larger variation as compared with paraxial corneal power. This is mostly due to the fact that raytracing additionally considers asphericity and aperture size and these can introduce additional variations.

Figure 2 shows the data for the corneal power values displayed as boxplots for the keratometry and paraxial calculations (upper graph) and for the raytracing calculations (lower graph) for Pup and for variations of the aperture size from 1 to 6 mm in steps of 1 mm. It is clear from the upper graph that corneal power using the Javal index is systematically higher compared with PKZ, and that PG and PFV are slightly lower compared with PKZ whereas PBV is quite similar to PKJ. The lower graph indicates that the raytracing power values PR and PW show a larger variation compared with the paraxial power values in the upper graph, and we see a systematic trend to higher power values with increasing aperture size. The raytracing power based on the measured pupil size is quite similar to the raytracing power with a 3 mm aperture size.

**FIGURE 3:**
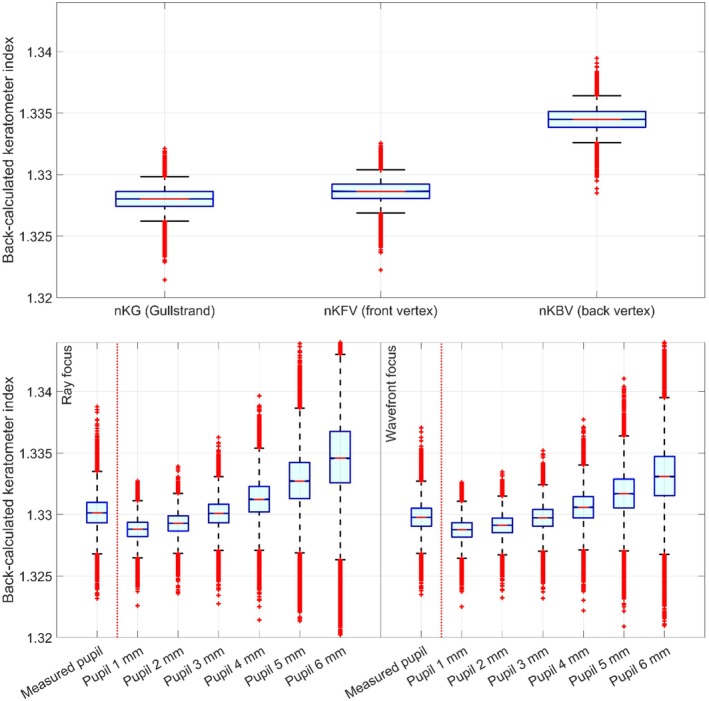
Keratometer index back‐calculated from corneal power values and the corneal front surface radius Ra based on the Copula expanded dataset (*N* = 30 000). Upper graph: Paraxial calculations based on a thick lens cornea model (nKG: From Gullstrand power PG referenced to the image‐sided principal plane, nKFV: From power referenced to the corneal front apex plane PFV, nKBV: From power referenced to the corneal back vertex plane PBV). Lower graph: Raytracing calculations (nKR: From ray focus, nKW: From wavefront focus) derived using the measured pupil size (Pup) and with apertures from 1 to 6 mm in steps of 1 mm. This graph indicates that the back‐calculated keratometer index increases with the aperture size as a result of the typically positive spherical aberration of the cornea. For similar reasons as in Figure 2, the back‐calculated keratometer index from raytracing generally shows a larger variation as compared with the paraxial calculations. For better visibility the *y*‐axis of both graphs has been truncated.

Figure 3 shows the data for the keratometer index back‐calculated from corneal power values and the corneal front surface radius displayed as boxplots for the paraxial calculations (upper graph) and for the raytracing calculations (lower graph) for Pup and for variations of the aperture size from 1 to 6 mm in steps of 1 mm (keratometry not shown in the graph as it uses a preset keratometer index). It is clear from the upper graph that nKG referenced to the image‐side principal plane yields slightly lower values compared with nKFV referenced to the front apex plane, and that nKBV yields systematically higher values as compared with NKB and nKFV. The lower graph again indicates that the back‐calculated keratometer index with raytracing power values nKR and nKW shows a systematically larger variation compared with nKG, nKFV, and nKBV especially with larger aperture sizes, and nKR and nKW systematically increase with larger aperture sizes.

Table 3 summarises the coefficients of the linear models for predicting the deviation of paraxial power values (PG, PFV and PBV) from PKZ (upper block) and the deviation of raytracing power values (PR and PW) from PKZ (lower block). Since the paraxial calculation does not consider asphericities or the aperture size we restricted the predictors to Ra, Rp, and CCT. However, for the raytracing calculation, we included as predictors Ra, Qa, Rp, Qp, and CCT. The aperture size is also included, as each Copula expanded datapoint was raytraced with the measured Pup as well as with apertures from 1 to 6 mm in steps of 1 mm (in total 210 000 raytracing calculations). For all models, the *F* statistics/*p*‐value as compared with a constant model was *F* > 0.11 × 10^−5^/*p* < 10^−25^.

**TABLE 3:** Upper block: Coefficients of the linear model for prediction of the difference of paraxial thick cornea power (PG: Gullstrand power, PFV: Thick cornea front vertex, PBV: Thick cornea back vertex) and keratometric power derived with the Zeiss keratometer index (PKZ), and coefficients of the linear model for prediction of the back‐calculated keratometer index.

Linear prediction model for the paraxial corneal power and back‐calculated keratometer indices							
From 30 000 Copula samples		Thick cornea Gullstrand		Thick cornea front vertex		Thick cornea back vertex	
~ 1 + Ra + Rp + CCT		PG‐PKZ	nKG	PFV‐PKZ	nKFV	PBV‐PKZ	nKBV
Prediction model coefficients	Intercept	−0.6385	1.3279	−0.4766	1.3285	1.0542	1.3343
	Ra	−0.7581	−0.0061	−0.7664	−0.0061	−0.9945	−0.0071
	Rp	0.9278	0.0071	0.9131	0.0070	0.9451	0.0072
	CCT	2.239 × 10^−4^	1.694 × 10^−6^	3.682 × 10^−4^	2.799 × 10^−6^	1.807 × 10^−3^	1.382 × 10^−5^

Linear prediction model for the raytracing corneal power and back‐calculated keratometer indices					
From 30 000 Copula samples each with 6 apertures (1 (1) 6 mm)		Raytracing ray focus		Raytracing wavefront focus	
~ 1 + Ra + Qa + Rp + Qp + CCT + Aperture		PR‐PKZ	nKR	PW‐PKZ	nKW
Prediction model coefficients	Intercept	0.5217	1.3336	0.26512	1.3323
	Ra	−0.9108	−0.0068	−0.8738	−0.0067
	Qa	1.1024	0.0084	0.8198	0.0063
	Rp	0.9279	0.0071	0.9242	0.0071
	Qp	−0.1787	−0.0014	−0.1333	−0.0010
	CCT	3.660 × 10^−4^	2.800 × 10^−6^	3.668 × 10^−4^	2.802 × 10^−6^
	Aperture	0.1536	0.0012	0.1143	8.757 × 10^−4^

*Note*: For the paraxial calculations, the anterior and posterior corneal radii Ra and Rp and the central corneal thickness CCT were used as predictors. Lower block: Coefficients of the linear model for prediction of the difference of raytracing cornea power (PR: From ray focus, PW: From wavefront focus) and PKZ, and the coefficients of the linear model for prediction of the back‐calculated keratometer index. For the raytracing calculations Ra, Rp, CCT, the asphericity of the front and back surface (Qa and QP) and the aperture size at the corneal front apex plane were used as predictors. Each Copula sample was raytraced with aperture sizes 1 (1) 6 mm.

**FIGURE 4:**
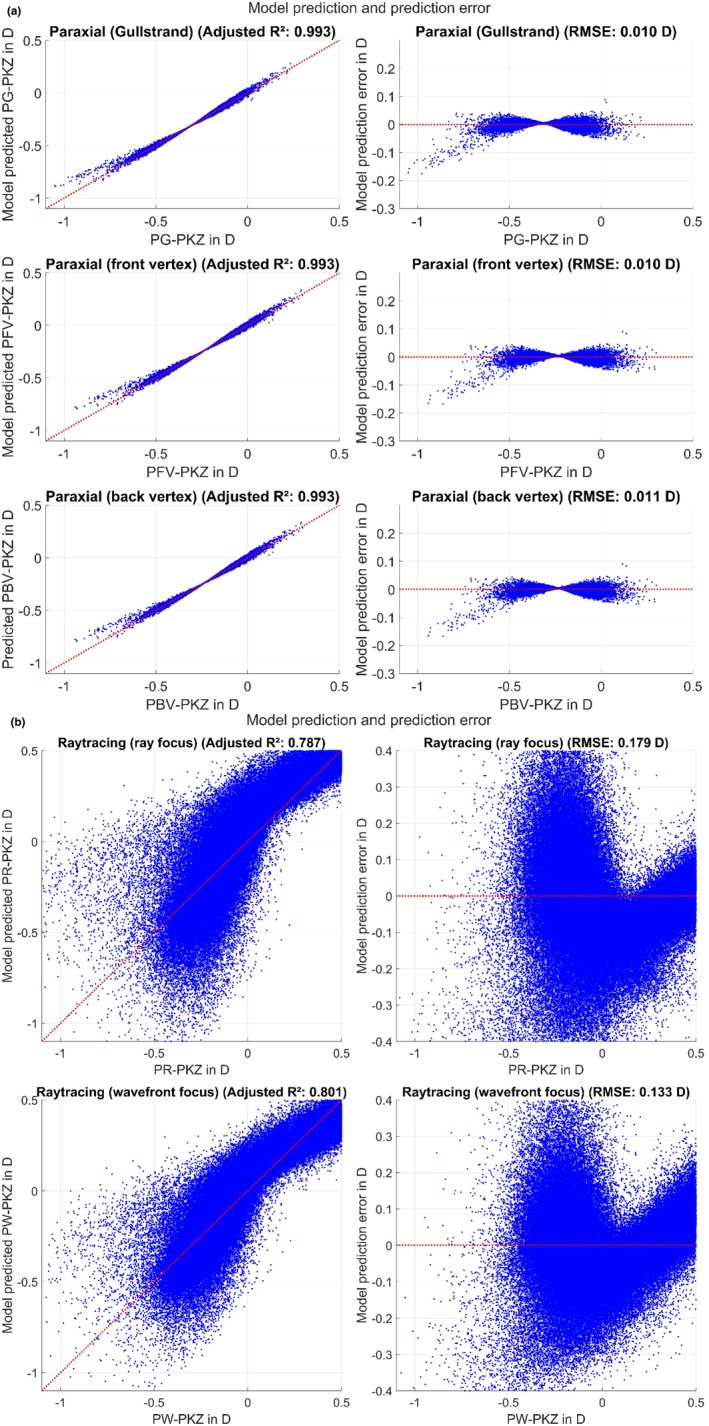
Scattergraphs showing the performance of the multivariable linear prediction models, with the observations on the *x*‐axis and the predictions (left graphs), or prediction errors (observation minus prediction, right graphs) on the *y*‐axis. (a) Performance of the linear models for the paraxial calculations (prediction of the deviation of Gullstrand power from keratometry (PG‐PKZ), power referenced to the front vertex plane (PFV‐PKZ), and power referenced to the back vertex plane (PBV‐PKZ)). (b) Performance of the linear models for the raytracing calculations (prediction of the deviation of raytracing power (ray focus) from keratometry (PR‐PKZ) and raytracing power (wavefront focus) from keratometry (PW‐PKZ)). The red lines on the graphs indicate a perfect match between observation and prediction. The adjusted *R* ^2^ values for the model and the root mean squared model prediction errors are detailed in the captions of the graphs.

Figure 4 shows the performance of the multivariable linear prediction models, with the observations on the *x*‐axis and the predictions (left graphs) or prediction errors (observation minus prediction, right graphs). In Figure 4a, the performance of the linear models is shown for the deviations of the paraxial calculations PG, PFV, and PBV from PKZ, and in Figure 4b, the performance of the linear models is shown for the deviations of the raytracing calculations PR and PW from PKZ. It is clear from the graphs that the models perform quite well in predicting the deviations of the paraxial power calculations from PKZ. However, the models do not perform well in predicting the deviations of the raytracing power calculations from PKZ.

## 4 DISCUSSION

There are several reasons why measuring the corneal front surface curvature with a keratometer and converting to corneal power using a keratometer index might be an unreliable strategy: (A) there is no unique keratometer index, and different keratometer indices yield different power values for the same Ra value (e.g. power values calculated using the Javal index typically exceed those derived using the Zeiss index by about 0.7 D). (B) in keratometry, it is necessary to use certain model assumptions for the corneal back surface properties and corneal thickness but these assumptions cannot be validated during keratometry. However, this article also illustrates that paraxial calculation of corneal power using a thick lens model is a large step forward in terms of generating realistic power data for the cornea. This approach includes both the corneal thickness and the curvatures of the corneal front and back surfaces, although it does not take account of the aperture or of the asphericity of the two corneal surfaces (Douthwaite, 2003; Langenbucher et al., 1998, 2011; Preussner et al., 2003; Sicam et al., 2006). If tomographic data for corneal radii and asphericities for both surfaces were available, together with corneal thickness and measures on the entrance pupil size, it would be possible to perform raytracing calculations to derive the location of the focus (Clement et al., 1987; Langenbucher et al., 2011; Langenbucher, Schrecker, et al., 2022; Langenbucher, Szentmáry, Weisensee, Cayless, et al., 2021; Langenbucher, Szentmáry, Weisensee, Wendelstein, et al., 2021). However, in raytracing applications the focus is quite undetermined as different metrics are used to define the best focus position (Langenbucher, Szentmáry, Weisensee, Wendelstein, et al., 2021). In this article, we used two different metrics to determine the focus position: the ray focus, which is defined as a plane perpendicular to the chief ray where the ray scatter is minimal, and the wavefront focus, where the optical path length differences of all rays are minimal (Langenbucher, Szentmáry, Weisensee, Wendelstein, et al., 2021). As we see from Table 2b and Figure 3, these two focus metrics yield slightly different results for our dataset. As the cornea typically shows some positive spherical aberration (Liou & Brennan, 1997; Navarro et al., 2006, 2019; Sicam et al., 2006), marginal rays are more refracted than paraxial rays, and as a consequence, both the raytracing corneal power and the back‐calculated keratometer index increase for larger aperture sizes. However, we have to be aware that paraxial calculation with a thick lens model requires reliable measures of the corneal back surface curvature and corneal thickness, and that raytracing additionally requires reliable measures on the asphericity of both surfaces (Schröder et al., 2018). Furthermore, depending on the measurement technique, we know that while the curvature of the corneal back surface and corneal thickness are quite reliable data, the asphericity of the corneal front surface and especially of the corneal back surface shows a larger variation (e.g. in repeat measurements) making raytracing results somewhat unpredictable (Schröder et al., 2018). Careful use of artificial tears could assist the measurement under certain conditions and could enhance the reproducibility or repeatability of corneal curvature and asphericity measurements (Schug et al., 2025). The raytracing results pretty much match the paraxial power data PFV (or PG) when the aperture size is small (e.g. 1 or 2 mm), but for larger apertures the spherical aberration has an increasing impact on corneal imaging and the back‐calculated keratometer index shows more and more variation (Langenbucher, Szentmáry, Weisensee, Wendelstein, et al., 2021).

For our study, we used a dataset with measurements from the Casia 2 anterior segment tomographer performed before cataract surgery. This means that the study population is not necessarily representative of young and healthy individuals (Consejo et al., 2021). Measurements which were potentially performed in pharmacological mydriasis (Pup >4.6 mm) were discarded as we feel that the fixation axis could be somewhat displaced compared with a physiological pupil due to a shift of the pupil centre (Consejo et al., 2021; Douthwaite, 2003; Preussner et al., 2003; Schneider et al., 2009; Schröder et al., 2018).

Copulas were used in this Monte Carlo simulation to expand our dataset (Chen et al., 2023; Choi & Seo, 2022). This technique is widely used in other disciplines such as physics and engineering as it maintains both the individual univariate distributions of all variables and the interactions between them (Langenbucher, Schrecker, et al., 2022). Our dataset of *N* = 4604 measurements was transformed into a uniform multivariate distribution (using the cumulative kernel distribution functions), and the interactions extracted using Kendall correlations. We then constructed a multivariate uniformly distributed Copula with *N* = 30 000 entries based on Kendall's τ correlation coefficients (Chen et al., 2023; Choi & Seo, 2022), and transformed back to the original space using the reversed cumulative kernel distribution functions. As a consequence, this Copula expanded dataset matches the original dataset in both the univariate distributions of all variables, and in the interactions (Chen et al., 2023; Choi & Seo, 2022; Langenbucher, Schrecker, et al., 2022). In general, as we have to cover the entire parameter space of ‘realistic’ parameter combinations for a Monte Carlo simulation for defining prediction models, the exact composition of the dataset is not the most critical part of this study (Choi & Seo, 2022; Preussner et al., 2003).

To keep the models simple for implementation in any consumer software, such as Excel, we restricted the modelling to multivariable linear models without interactions (Chen et al., 2023; Langenbucher, Szentmáry, et al., 2022). Instead of the paraxial or raytracing power itself, we decided to predict the deviation of the paraxial or raytracing power from the keratometric power PKZ. To avoid unnecessary complexity of the paper, we restricted our models to the deviation of calculated corneal power values from keratometry derived with the Zeiss keratometer index as we noticed that PFV as well as PR and PW with the measured pupil size Pup matched much better to PKZ than to PKJ. From Figure 4 we understand that our linear models for predicting PG‐PKZ, PFV‐PKZ, and PBV‐PKZ perform pretty well, having an RMS prediction error of about 0.01 D. However, our linear models for predicting PR‐PKZ or PW‐PKZ are not convincing, and the RMS prediction error (0.179 and 0.133 D) is more than 10 times larger compared with the corresponding models for paraxial calculations. This could be due to the fact that with raytracing we consider a number of factors in addition to the corneal front surface curvature, specifically the corneal back surface curvature, corneal thickness, the asphericities of both surfaces and the aperture size. We argue that in this situation, we would have to include mixed terms for interactions of the parameters, and probably higher order terms (quadratic or cubic terms) to improve the performance. However, including interaction and higher order terms bears the risk of systematic overfitting (Chen et al., 2023; Choi & Seo, 2022; Langenbucher, Szentmáry, Weisensee, Wendelstein, et al., 2021), and might make implementation in standard consumer software more difficult. We therefore recommend using raytracing techniques to extract the raytracing corneal power directly, instead of using prediction models.

However, our study shows some limitations: (1) We included measurements of the Casia2 from a population before cataract surgery. This means that the results and prediction models might not fully represent the situations of young and healthy eyes (de Sanctis et al., 2014). However, this effect may be minimal because the constitution of the study population does not seem to be critical in Monte Carlo modelling, provided we properly cover the entire parameter space. Further, we argue that the results might be slightly different for other Scheimpflug or optical coherence‐based tomographers. (2) We extracted corneal radius and asphericity data together with the corneal thickness and pupil size and restricted the raytracing to two dimensions. This means that we did not consider corneal astigmatism for the corneal front and back surfaces or a tilt of the visual axis, instead using a coaxially aligned optical model for our calculations. However, for area correction, we adjusted the ray density to simulate equally spaced rays over the entrance pupil (Langenbucher, Szentmáry, Weisensee, Wendelstein, et al., 2021), and with 3D raytracing additional decentration and tilt effects (Atchison et al., 2016; Clement et al., 1987) might complicate the interpretation of the results. Also, mirroring of the measurements for left or right eyes with respect to the facial axis or separate models for left and right eyes would be necessary. (3) We restricted the study to linear multivariable prediction models without interactions (Chen et al., 2023). The model performance might be better if interaction terms and/or higher order terms were included, especially for the prediction models for raytracing calculations (both for power and keratometer index), but implementation in consumer software might be complicated.

In conclusion, this Monte Carlo study based on a dataset containing anterior segment optical coherence measurements from a cataractous population shows that paraxial corneal power derived from a thick cornea model considering corneal front and back surface curvature and corneal thickness could yield corneal power values which systematically deviate from the keratometric corneal power as derived from corneal front surface radius converted to refractive power using a keratometer index, and also yields back‐calculated keratometer index values which systematically deviate from the standard values, especially as a function of pupil size. The results underline that raytracing corneal power derived from corneal front and back surface curvature and asphericity plus corneal thickness and an aperture size could yield corneal power values or back‐calculated keratometer index values which deviate even more from keratometric corneal power. While the paraxial corneal power could be predicted easily and accurately from corneal front and back surface curvature and corneal thickness using a simple linear model, the raytracing power is not fully amenable to prediction using linear models and we should use either direct raytracing calculation or prediction models with interaction or higher order terms to predict the raytracing power.

## CONFLICT OF INTEREST STATEMENT

The authors report no conflicts of interest and have no proprietary interest in any of the materials mentioned in this article. The authors received no specific funding for this work. Prof. Langenbucher reports speaker fees from Bausch and Lomb and Johnson & Johnson Vision outside the submitted work. Prof. Szentmáry and Dr. Cayless report no conflicts of interest. Dr. Hoffmann reports speaker fees from Heidelberg Engineering, Hoya Surgical and Johnson & Johnson outside the submitted work. Dr. Wendelstein reports research grants from Carl Zeiss Meditec AG, speaker fees from Carl Zeiss Meditec AG, Alcon, Rayner, Bausch and Lomb, and Johnson & Johnson Vision outside of the submitted work.

## References

[aos70052-bib-0001] Atchison, D.A. & Cooke, D.L. (2024) Refractive errors occurring with tilt of intraocular lenses. Ophthalmic & Physiological Optics, 44(1), 177–181. Available from: 10.1111/opo.13249 37962250

[aos70052-bib-0002] Atchison, D.A. , Suheimat, M. , Mathur, A. , Lister, L.J. & Rozema, J. (2016) Anterior corneal, posterior corneal, and lenticular contributions to ocular aberrations. Investigative Ophthalmology & Visual Science, 57(13), 5263–5270. Available from: 10.1167/iovs.16-20067 27701637

[aos70052-bib-0003] Chen, S. , Li, Q. , Wang, Q. & Zhang, Y.Y. (2023) Multivariate models of commodity futures markets: a dynamic copula approach. Empirical Economics, 12, 1–21. Available from: 10.1007/s00181-023-02373-2 PMC9924215 36818146

[aos70052-bib-0004] Choi, J.Y. & Seo, J. (2022) Copula‐based redundancy analysis. Multivariate Behavioral Research, 57(6), 1007–1026. Available from: 10.1080/00273171.2021.1941729 34310222

[aos70052-bib-0005] Clement, R.A. , Dunne, M.C. & Barnes, D.A. (1987) A method for raytracing through schematic eyes with off‐axis components. Ophthalmic and Physiological Optics, 7(2), 149–152. Available from: 10.1111/j.1475-1313.1987.tb01011.x 3658438

[aos70052-bib-0006] Consejo, A. , Fathy, A. , Lopes, B.T. , Ambrósio, R., Jr. & Abass, A. (2021) Effect of corneal tilt on the determination of Asphericity. Sensors (Basel), 21(22), 7636. Available from: 10.3390/s21227636 34833714 PMC8618126

[aos70052-bib-0007] de Sanctis, U. , Vinai, L. , Bartoli, E. , Donna, P. & Grignolo, F. (2014) Total spherical aberration of the cornea in patients with cataract. Optometry and Vision Science, 91(10), 1251–1258. Available from: 10.1097/OPX.0000000000000380 25192433

[aos70052-bib-0008] Douthwaite, W.A. (2003) The asphericity, curvature and tilt of the human cornea measured using a videokeratoscope. Ophthalmic & Physiological Optics, 23(2), 141–150. Available from: 10.1046/j.1475-1313.2003.00100.x 12641702

[aos70052-bib-0009] Iskander, D.R. , Collins, M.J. & Davis, B. (2001) Optimal modeling of corneal surfaces with Zernike polynomials. IEEE Transactions on Biomedical Engineering, 48(1), 87–95. Available from: 10.1109/10.900255 11235595

[aos70052-bib-0010] Langenbucher, A. , Eppig, T. , Seitz, B. & Janunts, E. (2011) Customized aspheric IOL design by raytracing through the eye containing quadric surfaces. Current Eye Research, 36(7), 637–646. Available from: 10.3109/02713683.2011.577265 21599465

[aos70052-bib-0011] Langenbucher, A. , Schrecker, J. , Eppig, T. , Schröder, S. , Cayless, A. , Schwemm, M. et al. (2022) Ratio of torus and equivalent power to refractive cylinder and spherical equivalent in phakic lenses ‐ a Monte‐Carlo simulation study. Acta Ophthalmologica, 100(1), 58–67. Available from: 10.1111/aos.14902 34018315

[aos70052-bib-0012] Langenbucher, A. , Seitz, B. , Kus, M.M. , Vilchis, E. & Küchle, M. (1998) Ellipsoidal fitting of corneal topography data after arcuate keratotomies with compression sutures. Ophthalmic Surgery and Lasers, 29(9), 738–748. 9760610

[aos70052-bib-0013] Langenbucher, A. , Szentmáry, N. , Cayless, A. , Münninghoff, L. , Wortmann, R. , Wendelstein, J. et al. (2022) Translation model for anterior segment tomographic data to corneal spherical aberration derived from a Monte‐Carlo simulation based on raytracing. Acta Ophthalmologica, 100(8), e1665–e1674. Available from: 10.1111/aos.15125 35233935

[aos70052-bib-0014] Langenbucher, A. , Szentmáry, N. , Weisensee, J. , Cayless, A. , Menapace, R. & Hoffmann, P. (2021) Back‐calculation of keratometer index based on OCT data and raytracing ‐ a Monte Carlo simulation. Acta Ophthalmologica, 99(8), 843–849. Available from: 10.1111/aos.14794 33576147

[aos70052-bib-0015] Langenbucher, A. , Szentmáry, N. , Weisensee, J. , Wendelstein, J. , Cayless, A. , Menapace, R. et al. (2021) Prediction model for best focus, power, and spherical aberration of the cornea: raytracing on a large dataset of OCT data. PLoS One, 16(2), e0247048. Available from: 10.1371/journal.pone.0247048 33617531 PMC7899355

[aos70052-bib-0016] Liou, H.L. & Brennan, N.A. (1997) Anatomically accurate, finite model eye for optical modeling. Journal of the Optical Society of America. A, Optics, Image Science, and Vision, 14(8), 1684–1695. Available from: 10.1364/josaa.14.001684 9248060

[aos70052-bib-0017] Navarro, R. , González, L. & Hernández, J.L. (2006) Optics of the average normal cornea from general and canonical representations of its surface topography. Journal of the Optical Society of America. A, Optics, Image Science, and Vision, 23, 219–232. Available from: 10.1364/josaa.23.000219 16477826

[aos70052-bib-0018] Navarro, R. , Rozema, J.J. , Emamian, M.H. , Hashemi, H. & Fotouhi, A. (2019) Average biometry of the cornea in a large population of Iranian school children. Journal of the Optical Society of America. A, Optics, Image Science, and Vision, 36(4), B85–B92. Available from: 10.1364/JOSAA.36.000B85 31044964

[aos70052-bib-0019] Preussner, P.R. , Wahl, J. & Kramann, C. (2003) Corneal model. Journal of Cataract and Refractive Surgery, 29(3), 471–477. Available from: 10.1016/s0886-3350(02)01512-2 12663008

[aos70052-bib-0020] Schneider, M. , Iskander, D.R. & Collins, M.J. (2009) Modeling corneal surfaces with rational functions for high‐speed videokeratoscopy data compression. IEEE Transactions on Biomedical Engineering, 56(2), 493–499. Available from: 10.1109/TBME.2008.2006019 19272911

[aos70052-bib-0021] Schröder, S. , Mäurer, S. , Eppig, T. , Seitzs, B. , Rubly, K. & Langenbucher, A. (2018) Comparison of corneal tomography. Repeatability, precision, misalignment, mean elevation, and mean pachymetry. Current Eye Research, 43, 709–716. Available from: 10.1080/02713683.2018.1441873 29482368

[aos70052-bib-0022] Schug, T. , Kohnen, T. , Kaiser, K.P. & Lwowski, C. (2025) Influence of artificial tears on corneal parameter measurement using three different devices: keratometry and Scheimpflug technology, a randomized trial. Acta Ophthalmologica, 103(5), e310–e317. Available from: 10.1111/aos.17487 40153153 PMC12235682

[aos70052-bib-0023] Sicam, V.A. , Dubbelman, M. & van der Heijde, R.G. (2006) Spherical aberration of the anterior and posterior surfaces of the human cornea. Journal of the Optical Society of America. A, Optics, Image Science, and Vision, 23(3), 544–549. Available from: 10.1364/josaa.23.000544 16539049

